# Transplantation of human endometrial perivascular cells with elevated CYR61 expression induces angiogenesis and promotes repair of a full-thickness uterine injury in rat

**DOI:** 10.1186/s13287-019-1272-3

**Published:** 2019-06-18

**Authors:** Zhongxun Li, Guijun Yan, Qiang Diao, Fei Yu, Xin’an Li, Xiaoqiang Sheng, Yong Liu, Yimin Dai, Huaijun Zhou, Xin Zhen, Yali Hu, Bruno Péault, Lijun Ding, Haixiang Sun, Hairong Li

**Affiliations:** 1 Department of Histology and Embryology of Shanxi Medical University, Taiyuan, 030001 China; 2 Center for Reproductive Medicine, Department of Obstetrics and Gynecology, the Affiliated Drum Tower Hospital of Nanjing University Medical School, Nanjing, 210008 China; 3 Department of Medical Imaging, Jinling Hospital, Nanjing University Medical School, Nanjing, 210002 China; 4 Center for Experimental Animal, the Affiliated Drum Tower Hospital of Nanjing University Medical School, Nanjing, 210008 China; 5 Department of Obstetrics and Gynecology, the Affiliated Drum Tower Hospital of Nanjing University Medical School, Nanjing, 210008 China; 6 Department of Experimental Medicine, the Affiliated Drum Tower Hospital of Nanjing University Medical School, Nanjing, 210008 China; 7 UKMRC Center for Regenerative Medicine and Center for Cardiovascular Science, University of Edinburgh, Edinburgh, Scotland, UK; 8 Clinical Center for Stem Cell Research, the Affiliated Drum Tower Hospital of Nanjing University Medical School, Nanjing, 210008 China; 9 Key Laboratory of Pharmaceutical Biotechnology, Nanjing University, Nanjing, China

**Keywords:** Endometrial perivascular cells, CYR61, Uterine injury, Neovascularization, Fertility

## Abstract

**Background:**

Disruptions of angiogenesis can have a significant effect on the healing of uterine scars. Human endometrial perivascular cells (CD146+PDGFRβ+) function as stem cells in the endometrium. Cysteine-rich angiogenic inducer 61 (CYR61) plays an important role in vascular development. The purpose of this study was to observe the effects of the transplantation of human endometrial perivascular cells (En-PSCs) overexpressing CYR61 on structural and functional regeneration in rat models of partial full-thickness uterine excision.

**Methods:**

We first sorted human En-PSCs from endometrial single-cell suspensions by flow cytometry. Human En-PSCs expressing low or high levels of CYR61 were then generated via transfection with a CYR61-specific small interfering ribonucleic acid (si-CYR61) construct or overexpression plasmid. To establish a rat model of uterine injury, a subset of uterine wall was then resected from each uterine horn in experimental animals. Female rats were randomly assigned to five groups, including a sham-operated group and four repair groups that received either PBS loaded on a collagen scaffold (collagen/PBS), En-PSCs loaded on a collagen scaffold (collagen/En-PSCs), En-PSCs with low CYR61 expression loaded on a collagen scaffold (collagen/si-CYR61 En-PSCs), and En-PSCs overexpressing CYR61 loaded on a collagen scaffold (collagen/ov-CYR61 En-PSCs). These indicated constructs were sutured in the injured uterine area to replace the excised segment. On days 30 and 90 after transplantation, a subset of rats in each group was sacrificed, and uterine tissue was recovered and serially sectioned. Hematoxylin and eosin staining and immunohistochemical staining were then performed. Finally, the remaining rats of each group were mated with fertile male rats on day 90 for a 2-week period.

**Results:**

Sorted En-PSCs expressed all recognized markers of mesenchymal stem cells (MSCs), including CD10, CD13, CD44, CD73, CD90, and CD105, and exhibited differentiation potential toward adipocytes, osteoblasts, and neuron-like cells. Compared with En-PSCs and En-PSCs with low CYR61 expression, En-PSCs with elevated CYR61 expression enhanced angiogenesis by in vitro co-culture assays. At day 90 after transplantation, blood vessel density in the collagen/ov-CYR61 En-PSCs group (11.667 ± 1.287) was greater than that in the collagen/En-PSCs group (7.167 ± 0.672) (*P* < 0.05) and the collagen/si-CYR61 En-PSCs group (3.750 ± 0.906) (*P* < 0.0001). Pregnancy rates differed among groups, from 40% in the collagen/PBS group to 80% in the collagen/En-PSCs group, 12.5% in the collagen/si-CYR61 En-PSCs group, and 80% in the collagen/ov-CYR61 En-PSCs group. In addition, four embryos were evident in the injured uterine horns of the collagen/ov-CYR61 En-PSCs group, while no embryos were identified in the injured uterine horns of the collagen/PBS group.

**Conclusions:**

The results showed that CYR61 plays an important role in angiogenesis. Collagen/ov-CYR61 En-PSCs promoted endometrial and myometrial regeneration and induced neovascular regeneration in injured rat uteri. The pregnancy rate of rats treated with transplantation of collagen/En-PSCs or collagen/ov-CYR61 En-PSCs was improved. Moreover, the number of embryos implantation on the injured area in uterus was increased after transplantation of collagen/ov-CYR61 En-PSCs.

**Electronic supplementary material:**

The online version of this article (10.1186/s13287-019-1272-3) contains supplementary material, which is available to authorized users.

## Background

Asherman syndrome can occur following uterine curettage, instrumentation, or infections in the pregnant or recently pregnant uterine cavity. It is often accompanied by scarring of the endometrium, hypomenorrhea, and abortion [1]. A previous study revealed that among women whose implantation sites were close to or crossing a scarred region, 55% or 88%, respectively suffered a spontaneous abortion, as compared with just 22% of women with implantation sites away from the scarred region. Indeed, the presence of such scarring reduces the likelihood of successful embryo implantation when the implantation site is close to the scar [2]. In a 10-year study including 638 women with Asherman syndrome, the chance of spontaneous recurrence of adhesion was 20.8% even when the initial grade of adhesions was grade 1 [3]. Surgical management via hysteroscopic lysis of adhesions is major and an effective treatment for uterine scars, but there are currently no effective means for preventing recurrence [4]. The microvascular density in patients with Asherman syndrome who respond to surgery is significantly increased as compared with that in patients who do not respond to surgery, suggesting that angiogenesis in the scar regions may affect endometrial repair [5]. Previous study using mesenchymal stem cells (MSCs) to repair the full-thickness injury in rat uterus has suggested that angiogenesis can be enhanced at a certain extent [6].

The human endometrium has a remarkable regenerative capacity and can grow 4–6 mm within 5–6 days after onset of menstruation [7]. In 2004, the clonogenicity of human endometrial epithelial and stromal cells was first identified [8]. In 2007, functional evidence for the existence of human and mouse endometrial stem cells was further discussed [9]. Subsequently, the markers CD146 and PDGFRβ were used to identify the endometrial stem cells. While these markers identify a perivascular region in both the functionalis and basalis of the human endometrium [10, 11], they are also expressed by perivascular cells, indicating that perivascular cells are closely related to endometrial mesenchymal stem cells (eMSCs) [10, 12, 13].

Perivascular cells are present at intervals along the walls of capillaries [14], have myogenic potential, exhibit migratory ability, and give rise to MSCs [12]. Perivascular cells are multipotent and can differentiate into chondrocytes, adipocytes, phagocytes, osteoblasts, and granulocytes. They express MSCs markers such as CD105, CD73, CD90, and CD44 [15]. Given their key properties, it is thus possible that endometrial perivascular cells (En-PSCs) may have a superior ability to regenerate scarred uterine tissue [16–19].

Cysteine-rich angiogenic inducer 61 (CYR61), also known as CCN family member 1 (CCN1), encoded by the *CYR61* gene, is a matricellular protein [20] that is highly expressed in endothelial cells and in smooth muscle cells throughout development and is essential for vascular integrity [21]. CYR61 is essential for cardiovascular development during embryogenesis [22]. In vitro, CYR61 promotes the migration of endothelial cells and stimulates the release of angiogenic factors from the extracellular matrix (ECM), which may contribute to the overall process of neovascularization in vivo [23]. CYR61 is also involved in angiogenesis and tissue repair [22, 24], and perivascular cells-specific loss of CYR61 reduces angiogenic signals in the mouse model of oxygen-induced retinopathy [25].

In this context, the aim of this study was to construct En-PSCs expressing different levels of CYR61 and to observe their effects on healing and related outcomes in a rat model of full-thickness uterine injury. In this study, En-PSCs with elevated CYR61 expression loaded on a collagen scaffold induced increased angiogenesis and promote functional regeneration of injured rat uteri. Our findings indicated that En-PSCs with elevated CYR61 expression loaded on a collagen scaffold may support uterine tissue regeneration.

## Methods

### Isolation and culture of En-PSCs

Endometrial tissue samples were obtained from women attending the Center for Reproductive Medicine of Nanjing Drum Tower Hospital from June 2017 to June 2018. All samples were collected with the informed consent of the patients, and approval from the ethics committee was obtained for this study. The tissue was washed twice with sterile D-Hanks solution, the muscle layer was stripped away, and the tissue was then cut up and digested in a mixture of 1 mg/mL collagenase I, II, and IV and 40 μg/mL deoxyribonuclease. After digestion and centrifugation, red blood cells were lysed for 5 min and then the cells were resuspended in D-hanks solution at 10^6^/mL. Next, 10 μL of anti-CD45-APC-Cy7 (1:100; BD Biosciences, San Jose, CA, USA), anti-CD144-PerCP-Cy5.5 (1:100; BD Biosciences), anti-CD56-PE-Cy7 (1:100; BD Biosciences), anti-CD34-PE (1:100; BD Biosciences), and anti-CD146-FITC (1:100; BD Biosciences) was added to the cell suspensions at 4 °C for 15 min in the dark. Cells were then incubated for 15 min with DAPI (BD Biosciences) for dead cell exclusion. At the same time, the isotype control and blank control were set. Following this incubation, cells were washed twice with D-hanks solution and resuspended at a final volume of 500 μL before flow cytometry. En-PSCs, which were identified as CD146+CD34-CD45-CD56-CD144 cells [12], were sorted from these single-cell suspensions and then cultured in DMEM-F12 (Gibco, Grand Island, NY, USA) media supplemented with 10% fetal bovine serum (FBS; Gibco) and 10 ng/ml basic fibroblast growth factor (bFGF; Gibco).

### Immunofluorescence analysis

Expression of CD146, CD31, PDGFRβ, and α-smooth muscle actin (α-SMA) were detected in the endometrium by immunofluorescence staining. Frozen sections of fresh endometrium were fixed in 4% paraformaldehyde and stained with primary antibodies such as anti-CD146 antibody (ab75769, Abcam, Cambridge, MA, USA), anti-CD31 antibody (ab187377, Abcam), anti-PDGFRβ antibody (ab139406, Abcam), and anti-α-SMA antibody (M0851, Dako, Glostrup, Denmark) at 4 °C overnight. Secondary Alexa Fluor 594-conjugated donkey anti-rabbit IgG (1: 1000, Invitrogen, Grand Island, NY, USA), Alexa Fluor 488-conjugated donkey anti-mouse IgG (1: 1000, Invitrogen), Alexa Fluor 488-conjugated donkey anti-rabbit IgG (1: 1000, Invitrogen), or Alexa Fluor 594-conjugated donkey anti-mouse IgG (1: 1000, Invitrogen) were used to stain the tissue. The nuclei were then stained with DAPI (Sigma, St. Louis, MO, USA).

En-PSCs were fixed in 4% paraformaldehyde and stained with primary antibodies such as anti-PDGFRβ antibody (ab139406, Abcam), anti-NG2 antibody (ab83178, Abcam), and anti-α-SMA antibody (ab5694, Abcam) at 4 °C overnight. Secondary Alexa Fluor 488-conjugated goat anti-rabbit IgG (1: 1000, Invitrogen) or Alexa Fluor 594-conjugated goat anti-rabbit IgG (1: 1000, Invitrogen) were used to stain cells. The nuclei were then stained with DAPI (Sigma), and cells were imaged using a fluorescence confocal microscope (Leica, Wetzlar, Germany).

### Flow cytometric analysis

Cell surface antigens of En-PSCs (passage 6) were analyzed by flow cytometer (BD Biosciences). Single-cell suspensions were harvested in 0.2% FBS/PBS. The cells were then incubated for 30 min with PE-conjugated anti-rat CD34 (BD Pharmingen, San Diego, CA, USA), CD144 (BD Pharmingen), CD56 (BD Pharmingen), CD105 (BD Pharmingen), CD13 (BD Pharmingen), HLA-DR (BD Pharmingen), FITC-conjugated anti-rat CD146 (BD Pharmingen), CD73 (BD Pharmingen), CD90 (BD Pharmingen), CD44 (BD Pharmingen), and CD45 (BD Pharmingen) at 4 °C for 30 min. The cells were then analyzed via flow cytometer (Becton Dickinson, USA).

### Differentiation of En-PSCs

En-PSCs (passage 6) were assessed for their multipotency using adipogenic, osteogenic, and neural-like differentiation assays. The cells were seeded at a density of 2 × 10^4^/cm^2^ in 24-well plates. Growth media was replaced with the appropriate differentiation medium when cells reached 90% confluency. Adipogenic and osteogenic induction media (Gibco) were used for differentiation. After 30 days, cells were stained with oil red O (Sigma) or alizarin red S (Gibco) to identify lipid droplets or calcium deposition, respectively. For neural-like differentiation, pre-induction media containing 10^−7^ mol/L all-trans-retinoic acid (ATRA; Sigma) and 10 ng/ml bFGF was added to cells for 24 h followed by the addition of modified neuronal medium for 6 h. Neural-like differentiation was detected via immunofluorescent staining for neurofilament medium polypeptide (NF-M; 1:100, sc-16143, Santa Cruz Biotechnology, Santa Cruz, CA, USA) and neuron-specific enolase (NSE; 1:100, sc-292097, Santa Cruz Biotechnology).

### Liquid chromatography-tandem mass spectrometry (LC-MS/MS) analysis

En-PSCs were treated with phenol red-free and serum-free DMEM-F12 for 48 h, and then, the supernatant was collected. This supernatant was centrifuged at 4000×*g* for 20 min in a concentrating tube (UFC903001, Millipore, Bedford, MA, USA) to obtain concentrated supernatant. Protein in the concentrated supernatant was quantified via BCA Protein Assay (Thermo Fisher Scientific, Rockford, IL, USA). After polyacrylamide gel electrophoresis, Coomassie blue staining was performed, followed by decolorization with a decolorizing solution (10% Acetic acid, 45% Methanol, 45% ddH_2_O). Target strips were then cut out and analyzed by LTQ Orbitrap Velos Pro (Thermo Finnigan, CA, USA).

### Wound-healing assay

En-PSCs were treated with phenol red-free and serum-free DMEM-F12 for 48 h, and then, the supernatant was collected. This supernatant was centrifuged at 4000×*g* for 20 min in a concentrating tube (UFC903001, Millipore) to obtain concentrated supernatant. Wound-healing assay was carried out to detect the migration ability of the human endometrial stromal cells (ESCs) cultured with the concentrated supernatant. The percentage of cell migration was analyzed after 24 h treatment.

### Tube formation assays

First, liquid Matrigel (50 μl) (BD Biosciences) was plated per well of a 96-well plate at 4 °C and was then incubated for 30 min at 37 °C. Subsequently, human umbilical vein endothelial cells (HUVECs) (1 × 10^4^/well) suspended in serum-free M199 were added to each well. HUVECs were served as blank control. Vascular endothelial growth factor (VEGF; 0.3 nmol/L, 100–20, Peprotech, Rocky Hill, NJ, USA) was added as positive control. Recombinant human CYR61 (CYR61, C600220-005, Sangon Biotech, ShangHai, China) was used at a concentration of 40 ng/ml. Anti-CYR61 antibody (ab24448, Abcam) was added in order to block CYR61. These cells were then incubated for 2 h at 37 °C, and tube-like structures were photographed with an Olympus digital camera (magnification of × 100). The mean number of observed tubes was then counted in three wells, with three to five random fields per well.

### Small interfering RNA and plasmid transfection

When cultured En-PSCs reached 70–80% confluency, negative control small interfering ribonucleic acid (si-NC, 50 nM, RiboBiO, Guangzhou, China), CYR61-specific small interfering ribonucleic acid (si-CYR61, 50 nM, RiboBiO), vector control (GV141, 5 μg/10^6^ cells, GENE CHEM, ShangHai, China), or CYR61 overexpression plasmids (GV141-CYR61, 5 μg/10^6^ cells, GENE CHEM) were transfected into En-PSCs (passage 6) using the Lipofectamine® 3000 transfection reagent (L3000075, Invitrogen). En-PSCs were taken as a control group. The transfection efficiency among five groups, including control group, si-NC group, si-CYR61 group, vector group, and ov-CYR61 group, was detected after 48 h by western blotting and ELISA.

### Western blotting

Cells were washed with PBS, and 1 mL of cell lysis buffer (50.0 mmol/L Tris pH = 7.6, 150.0 mmol/L NaCl, 0.1% SDS, 1.0% NP-40, protease inhibitor cocktail) was added. Cells were then scraped on ice, and lysis was allowed to proceed at 4 °C for 30 min, followed by centrifugation at 15000 rpm for 15 min. Supernatant was then collected, and protein was quantified via BCA. Equal amounts (30 μg) of total protein were then subjected to standard Western blotting. Resultant PVDF membranes (Millipore) were probed with primary antibodies against CYR61 (1:500, ab230947, Abcam), β-actin (1: 10000, AP0060, Bioworld, St Louis Park, MN, USA), or Flag (1:10000, Sigma). Anti-rabbit IgG (1:10000, A0545, Sigma) was used as a secondary antibody. Detection was performed using an enhanced chemiluminescence kit (Amersham Biosciences Corp., Piscataway, NJ, USA).

### In vitro vascular network formation

Cell culture/co-culture experiments using Matrigel systems were performed to observe the capillary-like network formation. En-PSCs (5 × 10^3^) (above cells transfected for 48 h) or HUVECs (5 × 10^3^) were seeded onto Matrigel-coated well and incubated for 2 h. This experiment was divided into five groups including control group, si-NC group, si-CYR61 group, vector group, and ov-CYR61 group. Co-culture of HUVECs with En-PSCs was served as control. Tube-like structures were photographed with an Olympus digital camera (magnification of × 100). The mean number of observed tubes was then counted in three wells, with three to five random fields per well.

### Transwell migration assays

Transwell migration assays were divided into six groups including control group, si-NC group, si-CYR61 group, vector group, ov-CYR61 group, and CYR61 group. These experiments were conducted using 24-well plates containing Transwell inserts (Corning Incorporated, 8 μm, Corning, NY, USA) in vitro [26]. Briefly, En-PSCs (1 × 10^6^) (blank control), transfected En-PSCs, or CYR61 (40 ng/ml) were added in the lower chamber. Then, 1300 μl DMEM-F12 containing 10% FBS was added in the lower chamber, respectively. HUVECs (1 × 10^5^) (passage 3) were resuspended in 200 μl serum-free medium and placed in the upper chamber. After co-culture at 37 °C for 24 h, HUVECs on the membrane were scraped off and the migrating cells under membrane were observed using a microscope. The migrating cells were fixed with methanol and stained with 0.1% crystal violet for 20 min. The mean number of observed cells was then counted per well, with five random fields per well (magnification of × 400).

### Elisa

After 48 h, the supernatant (Phenol red-free medium) of transfected En-PSCs was collected. The supernatant of En-PSCs was served as control. CYR61 levels in the supernatant were measured via an Enzyme-linked immunosorbent assay (EK1203, BOSTER) according to the manufacturer’s instructions. The optical density value (OD value) was measured by a microplate reader (Thermo, MA, USA) at 450 nm.

### Proliferation assay

Cell-counting kit-8 (CCK-8) (Dojindo, Kumarmoto, Japan) was used to detect the cell proliferation. After 48 h, these En-PSCs, transfected negative control small interfering ribonucleic acid (si-NC, 50 nM), CYR61-specific small interfering ribonucleic acid (si-CYR61, 50 nM), GV141 control plasmid (vector, 5 μg/10^6^ cells), and GV141-CYR61 overexpression plasmids (ov-CYR61, 5 μg/10^6^ cells) were digested and seeded at 2 × 10^3^ cell per well into 96-cell plates, six parallel wells for each group, which were conventionally cultured in complete medium (100 μl/well) for 1 week.

The CCK-8 reagent (10 μl) was added in phenol red-free medium (100 μl) per well. After incubation for 2 h, the optical density value (OD value) was measured at 450 nm using a microplate reader (Thermo). The growth curve was draw based on the mean value of the eight counts in each group.

### Uterine horn injury model and En-PSCs transplantation

Uterine horn injury model was established according to our previous reports [6, 27, 28]. Briefly, animal experiments were conducted in accordance with the guidelines of the Experimental Animals Management Committee (Jiangsu Province, China). In total, 44 female Sprague-Dawley (SD) rats (250–280 g) were purchased from the Animal Model Center of Nanjing Medical University. Vaginal smears were obtained daily between 08:00–10:00 AM. Rats with consecutive 4-day estrous cycles were randomly assigned to five groups, including a sham-operated group (sham; 18 uterine horns), a PBS loaded on a collagen scaffold group (collagen/PBS; 18 uterine horns), an En-PSCs loaded on a collagen scaffold group (collagen/En-PSCs; 18 uterine horns), an En-PSCs with low CYR61 expression loaded on a collagen scaffold group (collagen/si-CYR61 En-PSCs; 16 uterine horns), and an En-PSCs overexpressing CYR61 loaded on a collagen scaffold group (collagen/ov-CYR61 En-PSCs; 18 uterine horns). After rats were anesthetized, uterine horns were exposed via a low abdominal midline incision and a region in the uterine wall of roughly 1.5 cm in length and 0.5 cm in width was resected, while the mesometrium side was retained. In the sham group, uterine horns were exposed but not resected. Collagen membrane (ZH-BIO, Yantai, China) was used as an absorbable scaffold material for En-PSCs delivery. The four different kinds of collagen scaffolds (collagen/PBS, collagen/si-CYR61 En-PSCs, collagen/En-PSCs, and collagen/ov-CYR61 En-PSCs), which were of a dimension matching the resected area, were then sutured into injured area to replace the excised segment. Following completion of the surgery, rats received intramuscular injections of penicillin twice a day for 3 days.

### Histological analysis

On days 30 and 90 post-transplantation, a subset of rats (*n* = 4 uterine horns) from each group was euthanized. The injured site of each uterine horn was dissected, fixed in neutral formaldehyde for 24 h, and embedded in paraffin perpendicularly. Sections (3 μm) of uterine horns were then prepared transversally.

Hematoxylin and eosin (H&E) staining was then employed to observe tissue structure. Sections were stained for immunohistochemistry with anti-α-smooth muscle actin antibody (α-SMA, 1:1500, ab5694, Abcam) and anti-von Willebrand factor antibody (vWF; 1:10000, ab6994, Abcam). The thickness of endometrium was measured using ImageJ (National Institutes of Health, USA). The percentage of α-SMA positive area (α-SMA positive area of the injured region/total α-SMA positive area) was used to evaluate smooth muscle abundance with the Image-Pro Plus software (Media Cybernetics, Inc., Rockville, MD, USA). Blood vessel density was evaluated in at least three randomly selected fields per section under a magnification of × 400 [6].

### Fertility test

Fertility testing was employed to assess the function of the injured uteri, to determine whether they were receptive to fertilized ova and to assess whether they were able to support embryos to a late stage of pregnancy. Ninety days post-surgery, rats in estrus (*n* = 8 uterine horns for the collagen/si-CYR61 En-PSCs group, and *n* = 10 uterine horns for the sham group, the collagen/PBS group, the collagen/En-PSCs group, the collagen/si-CYR61 En-PSCs group, or collagen/ov-CYR61 En-PSCs group) were mated with 10-week-old fertile male Sprague-Dawley rats. Day 0 of pregnancy was defined as the day a vaginal plug was found. Rats were then euthanized on gestation day 15–19, and uterine horns were examined for the presence of embryos.

### Statistical analysis

Data were present as means ± S.E.M. and were analyzed using IBM SPSS Statistics Package for Social Science (Version 22.0, IBM Corp., Armonk, NY, USA). Multiple group comparisons were determined by one-way ANOVA. The pregnancy rates were presented as count and percentage, and multiple group comparisons were analyzed by Fisher exact test followed by Bonferroni correction. *P* < 0.05 was the threshold of statistical significance.

## Results

### Human endometrial En-PSCs phenotypes

**Fig. 1:**
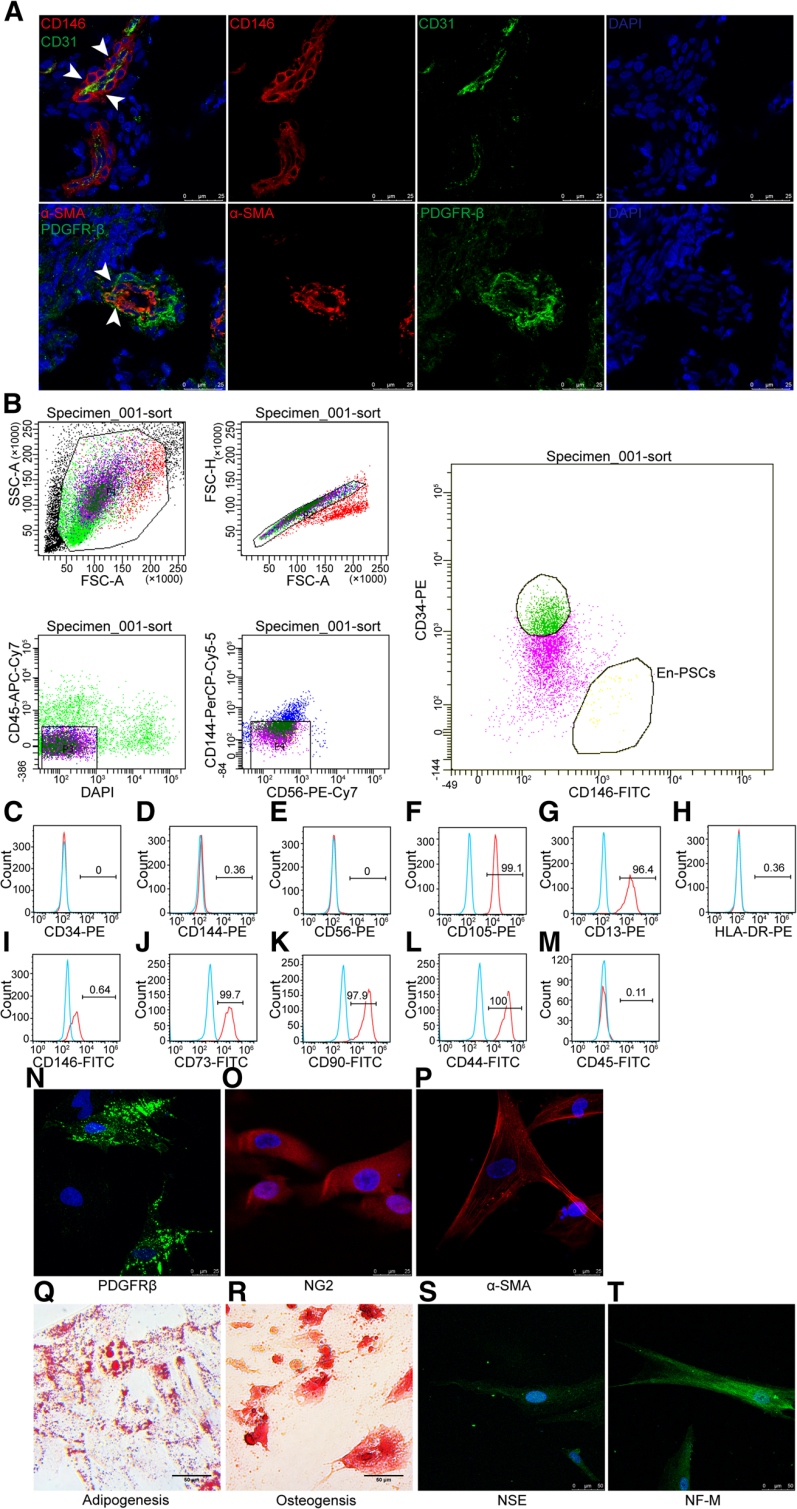
Phenotype and multidirectional differentiation potential of En-PSCs. **a** Immunofluorescence staining in human endometrium displayed CD146, CD31, PDGFRβ, and α-SMA. The arrow showed En-PSCs. Scale bars, 25 μm. **b** En-PSCs (CD146+CD34-CD45-CD56-CD144-) were sorted by flow cytometry. **c**–**m** Flow cytometry analysis of immune-markers in En-PSCs. **n**–**p** En-PSCs expressed PDGFRβ, NG2, and α-SMA. Scale bars, 25 μm. **q** Intracellular lipid droplet induced from En-PSCs were detected by Oil red O staining. Scale bars, 50 μm. **r** Calcified nodule stained with Alizarin red S indicated that En-PSCs differentiated to osteogenic lineage. Scale bars, 50 μm. Neural-like differentiation was confirmed by immunofluorescence staining with anti-NSE antibody (**s**) and anti-NF-M antibody (**t**). Scale bars, 50 μm

CD146, PDGFRβ, and α-SMA are the markers of perivascular cells, and CD31 is used to identify endothelial cells [29]. En-PSCs were detected around the blood vessels in human endometrium (Fig. 1a). Then, En-PSCs (CD146+CD34-CD45-CD56-CD144-) were sorted by flow cytometry from endometrial tissue samples (Fig. 1b). Percentage of immunophenotype in En-PSCs at passage 6 for CD34 was 0% (Fig. 1c), for CD144 was 0.36% (Fig. 1d), for CD56 was 0% (Fig. 1e), for CD105 was 99.1% (Fig. 1f), for CD13 was 96.4% (Fig. 1g), for HLA-DR was 0.36% (Fig. 1h), for CD146 was 64% (Fig. 1i), for CD73 was 99.7% (Fig. 1j), for CD90 was 97.9% (Fig. 1k), for CD44 was 100% (Fig. 1l), and for CD45 was 0.11% (Fig. 1m). These were consistent with previous reports of MSCs surface markers [28]. Cultured En-PSCs at passage 6 expressed PDGFRβ (Fig. 1n), NG2 (Fig. 1o), and α-SMA (Fig. 1p).

### Assessment of En-PSCs multipotency

To assess whether these isolated En-PSCs were capable of multilineage differentiation, the cells grown to passage 6 were cultured in media formulated to induce adipogenesis, osteogenesis, or neural-like differentiation. For adipogenesis, lipid droplets were detected by oil red O staining on day 30 (Fig. 1q). For osteogenesis, bone nodules were detected by alizarin red S staining on day 30 (Fig. 1r). Neural-like differentiation was confirmed by NSE (Fig. 1s) and NF-M (Fig. 1t) via immunofluorescence staining. These results suggested that En-PSCs had multilineage differentiation potential.

### Human En-PSCs secrete angiogenesis-related factors

A mass spectrometry analysis revealed En-PSCs secrete multiple pro-angiogenic factors, as well as CYR61 (Table 1). A total of 929 proteins were identified by mass spectrometry. A GO Functional Annotation Clustering analysis via DAVID 6.8 online (https://david.ncifcrf.gov/) suggested that there were 203 secreted proteins (Additional file 3: Table S3) among these identified proteins. These 203 secreted proteins were compared with 781 proteins known to be associated with angiogenesis (Human sapiens) in the Uniprot Online Protein Library (https://www.uniprot.org/), of which 38 total angiogenesis-related proteins were identified (Table 1).

**Table 1:** Angiogenesis-related protein in En-PSCs supernatant

Accession	Score	Content	Protein name	Function
P09486	618	12.3	BM-40	Negative regulation of Angiogenesis
P08253	3054	12.09	MMP-2	Angiogenesis
Q15582	1967	11.4	Beta ig-h3	Angiogenesis
P05121	1157	5.75	Plasminogen activator inhibitor 1	Angiogenesis
P07355	712	5.56	Annexin A2	Angiogenesis
P02751	5713	5.14	Fibronectin	Angiogenesis
P36955	985	3.22	PEDF	Negative regulation of angiogenesis
P08572	2285	2.9	Collagen alpha-2(IV) chain	Angiogenesis
Q16610	723	2.62	Extracellular matrix protein 1	Angiogenesis
P07585	385	2.04	Decorin	Negative regulation of angiogenesis
O60565	229	1.8	Gremlin-1	Cell migration involved in sprouting angiogenesis
Q99969	150	1.67	Retinoic acid receptor responder protein 2	Angiogenesis
P04083	372	1.66	Annexin A1	Positive regulation of cell migration involved in sprouting angiogenesis
Q08431	310	1.39	MFGM	Angiogenesis
Q92743	269	1.22	Serine protease HTRA1	Angiogenesis
P27658	197	0.93	Collagen alpha-1(VIII) chain	Angiogenesis
Q969H8	117	0.93	MYDGF	Angiogenesis
Q9Y6C2	540	0.93	EMILIN-1	Negative regulation of angiogenesis
Q9Y4K0	445	0.79	Lysyl oxidase homolog 2	Angiogenesis
P31151	92	0.69	Protein S100-A7	Angiogenesis
P02462	682	0.55	Collagen alpha-1(IV) chain	Blood vessel morphogenesis
Q15389	204	0.47	ANG-1	Sprouting angiogenesis
O15230	1412	0.36	Laminin subunit alpha-5	Angiogenesis
E9PEP6	196	0.29	Neuropilin	Angiogenesis
P98160	1080	0.23	HSPG	Angiogenesis
O60687	119	0.19	Sushi repeat-containing protein SRPX2	Angiogenesis
P29279	76	0.17	CTGF	Angiogenesis
O00622	95	0.16	CYR61	Intussusceptive angiogenesis, wound healing, spreading of cells
Q14118	109	0.14	Dystroglycan	Angiogenesis involved in wound healing
Q13443	58	0.11	ADAM 9	Angiogenesis
P39060	209	0.09	Collagen alpha-1(XVIII) chain	Angiogenesis
P36222	33	0.08	Chitinase-3-like protein 1	Positive regulation of angiogenesis
Q9GZP0	99	0.08	PDGF-D	Angiogenesis
P01024	73	0.07	Complement C3	Positive regulation of angiogenesis
O15123	34	0.06	ANG-2	Angiogenesis
O60462	32	0.06	Neuropilin-2	Angiogenesis
Q12884	38	0.04	Prolyl endopeptidase FAP	Angiogenesis

*MMP1* matrix metalloproteinase 1, *ANG-1* angiopoietin-1, *Beta ig-h3* transforming growth factor-beta-induced protein ig-h3, *PDGF-D* platelet-derived growth factor D, *CTGF* connective tissue growth factor

### En-PSCs with different CYR61 levels show different capacity for angiogenesis

**Fig. 2:**
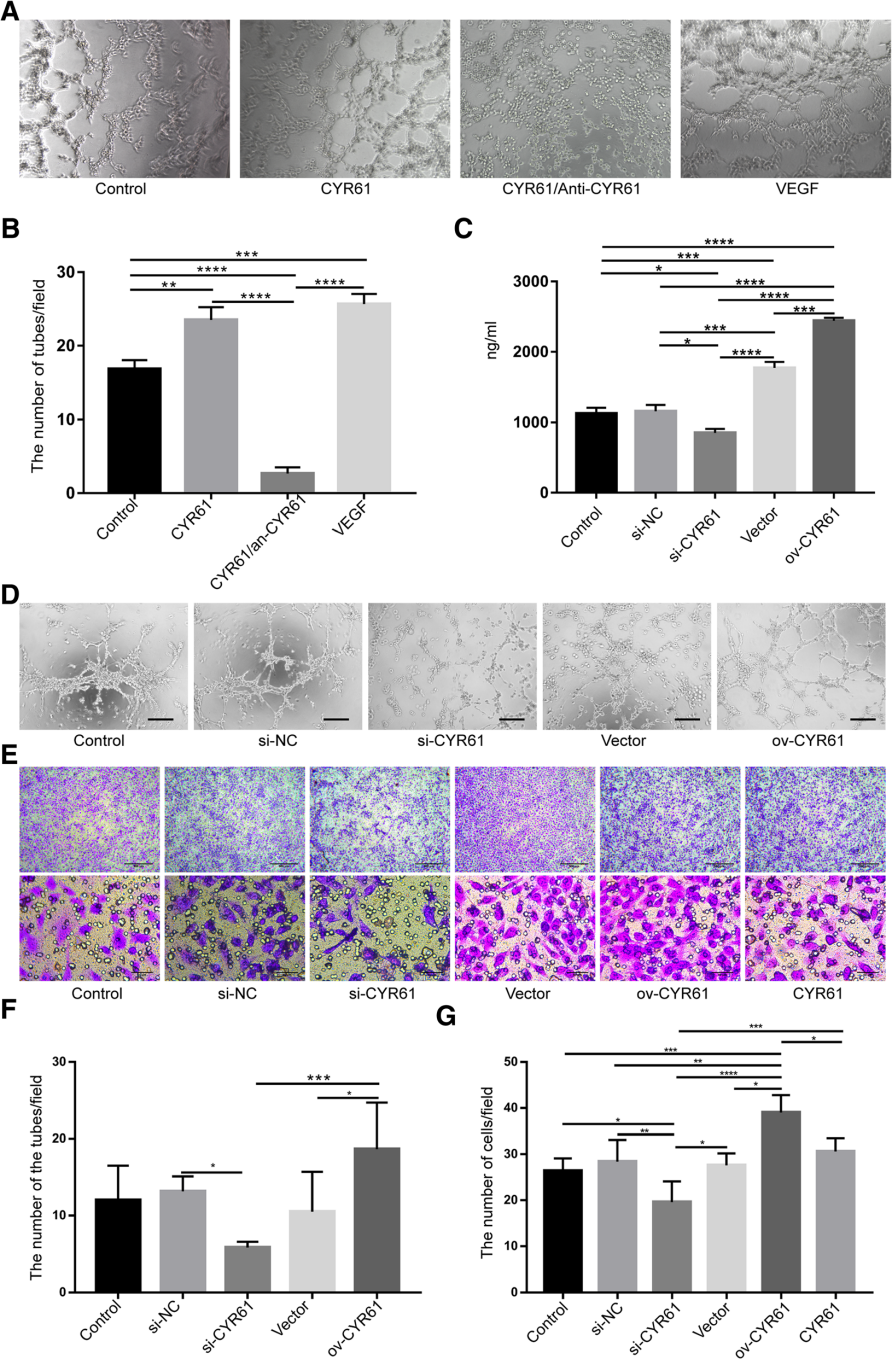
En-PSCs with elevated CYR61 expression induce angiogenesis. **a** The tube formation after treatment with CYR61 alone, combined CYR61&Anti-CYR61, VEGF alone, were microscopically compared. **b** The mean number of observed tubes was counted in three wells, with three to five random fields per well. **c** ELISA were performed 48 h after transfection of si-CYR61 or ov-CYR61. The concentration of CYR61 was measured in the supernatant of transfected En-PSCs. **d** Tube formation assays were performed 48 h after transfection of si-CYR61 or ov-CYR61. The tube formation after co-culture of HUVECs with transfected En-PSCs were microscopically compared. **f** The mean number of observed tubes was counted in three wells, with three to five random fields per well. Scale bars, 200 μm. **e** Effect of transfected En-PSCs on HUVECs migration. The HUVECs migrated to the bottom of the transwell membrane were stained with crystal violet. Scale bars, 500 μm. Local magnification of figures were showed in the pictures below. Scale bar, 50 μm. **g** The mean number of observed cells was then counted per well, with five random fields per well (magnification of × 400). Each experiment was repeated three times. Data were presented as mean + SEM. *P < 0.05, **P < 0.01, ***P < 0.001, and ****P < 0.0001

The role of CYR61 in angiogenesis was evaluated via a tube formation assay and an antibody blocking test. As shown in Fig. 2a, b, the number of tubes per field in wells treated with human recombinant CYR61 protein (23.500 ± 1.727) was significantly higher than that in control wells (16.830 ± 1.195) (*P* < 0.01) or in the group which received a CYR61 blocking antibody (2.667 ± 0.843) (*P* < 0.0001). Interestingly, the number of tubes per field in CYR61 blocking group was significantly lower than in the control group (*P* < 0.0001).

After transfection, different expression levels of CYR61 were confirmed in En-PSCs by Western Blotting (Additional file 1: Figure S1A). Secreted CYR61 levels were also measured in the culture supernatant by ELISA (Fig. 2c). CYR61 levels in the supernatant of the ov-CYR61 group (2442 ± 41.64 pg/ml) was nearly twice that of the control group (1127 ± 81.25 pg/ml) (*P* < 0.0001). The concentration of CYR61 in the si-CYR61 group (851.6 ± 57.03 pg/ml) was lower than that in the control group (*P* < 0.05).

Furthermore, CCK-8 assay was also performed to measure the differences of proliferative capability in control group, si-NC group, si-CYR61 group, vector group, and ov-CYR61 group, but no significant differences were observed (Additional file 1: Figure S1B). As shown in Fig. 2d, in vascular network formation assays in vitro, the number of tubes per field in the ov-CYR61 group (18.67 ± 2.472) was larger than that in the si-CYR61 group (5.833 ± 3.807) (*P* < 0.001). The number of tubes per field in si-NC group (13.17 ± 0.792) was also larger than that in si-CYR61 group (*P* < 0.001). In a migration assay (Fig. 2e), the number of cells per field in the ov-CYR61 group (39 ± 1.703) was greater than that in the si-CYR61 group (19.6 ± 2.015) (*P* < 0.0001) or the control group (26.4 ± 1.218) (*P* < 0.001). Additionally, the number of cells per field in the control group was greater than that in the si-CYR61 group (*P* < 0.05).

### Utilizing En-PSCs for the treatment of full-thickness uterine injury

At 30 days after surgical treatment, collagen scaffolds implanted into rats had been degraded. We found that the morphology and blood supply of rat uterine tissue in the collagen/ov-CYR61 En-PSCs group were better than those in the collagen/si-CYR61 En-PSCs group or the collagen/PBS group (Additional file 2: Figure S2).

**Fig. 3:**
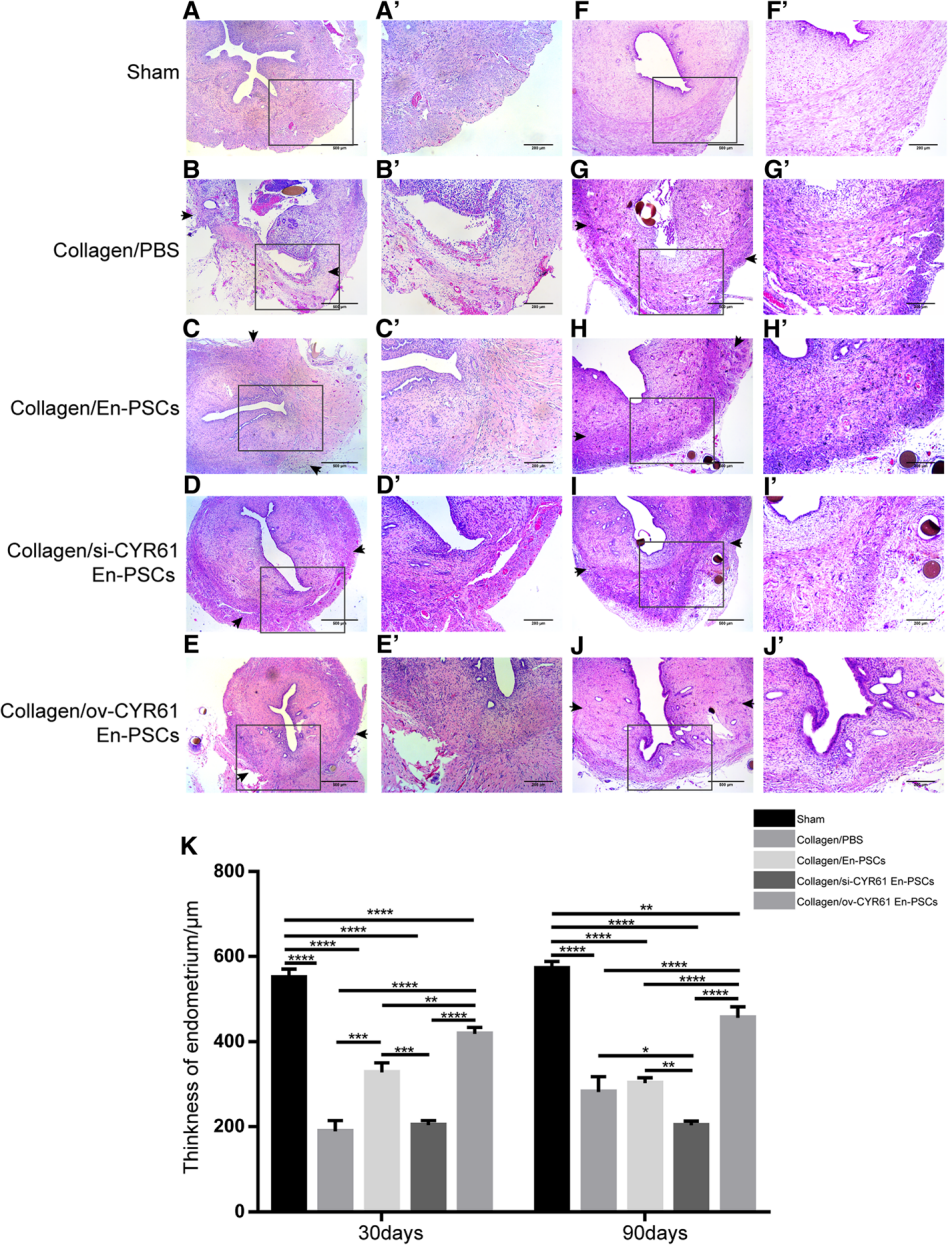
The regenerative uterine horns after collagen/transfected En-PSCs transplantation. Histological structures of the regenerative uterine horns at 30 days (**A**–**E**) and 90 days (**F**–**J**) in sham group (**A** and **F**), collagen/PBS group (**B** and **G**), collagen/En-PSCs group (**C** and **H**), collagen/si-CYR61 En-PSCs group (**D** and **I**), and collagen/ov-CYR61 En-PSCs group (**E** and **J**). Arrowheads indicated repair sites. Scale bar, 500 μm. Local magnification of figures (**A**–**J**) were showed in the pictures (**A′**–**J′**). Scale bar, 200 μm. **K** Statistical analysis of the thickness of endometrium in the five groups. Each experiment was repeated four times. Data were presented as mean ± SEM. *P < 0.05, **P < 0.01, ***P < 0.001, and ****P < 0.0001

Hematoxylin-eosin staining was next used to measure the endometrial thickness at the injury uterine sites. After 30 days of repair, relative to the collagen/PBS group, endometrial morphology was more complete in the three cell treatment groups (Fig. 3C–E, C′–E′). Endometrial thickness in the collagen/ov-CYR61 En-PSCs group (418.076 ± 15.685 μm) was greater than that in the collagen/PBS group (188.798 ± 25.703 μm) (*P* < 0.0001), the collagen/En-PSCs group (327.594 ± 22.990 μm) (*P* < 0.01), or the collagen/si-CYR61 En-PSCs group (203.755 ± 10.246 μm) (*P* < 0.0001) (Fig. 3K). In addition, endometrial thickness in the collagen/En-PSCs group was thicker than in the collagen/PBS group (*P* < 0.001) or the si-CYR61 En-PSCs group (*P* < 0.001) (Fig. 3K). After 90 days, endometrial thickness in the collagen/ov-CYR61 En-PSCs group (456.075 ± 26.535 μm) (Fig. 3J, J′) was greater than that in the collagen/PBS group (281.459 ± 35.876 μm) (*P* < 0.0001), the collagen/En-PSCs group (302.211 ± 12.466 μm) (*P* < 0.0001), or the collagen/si-CYR61 En-PSCs group (202.956 ± 10.095 μm) (*P* < 0.0001) (Fig. 3K). Indeed, En-PSC supernatant promote the migration of endometrial stromal cells (ESCs) in vitro (Additional file 1: Figure S1C), although there was no significant difference in the proliferation of ESCs in En-PSCs supernatant (Additional file 1: Figure S1E).

**Fig. 4:**
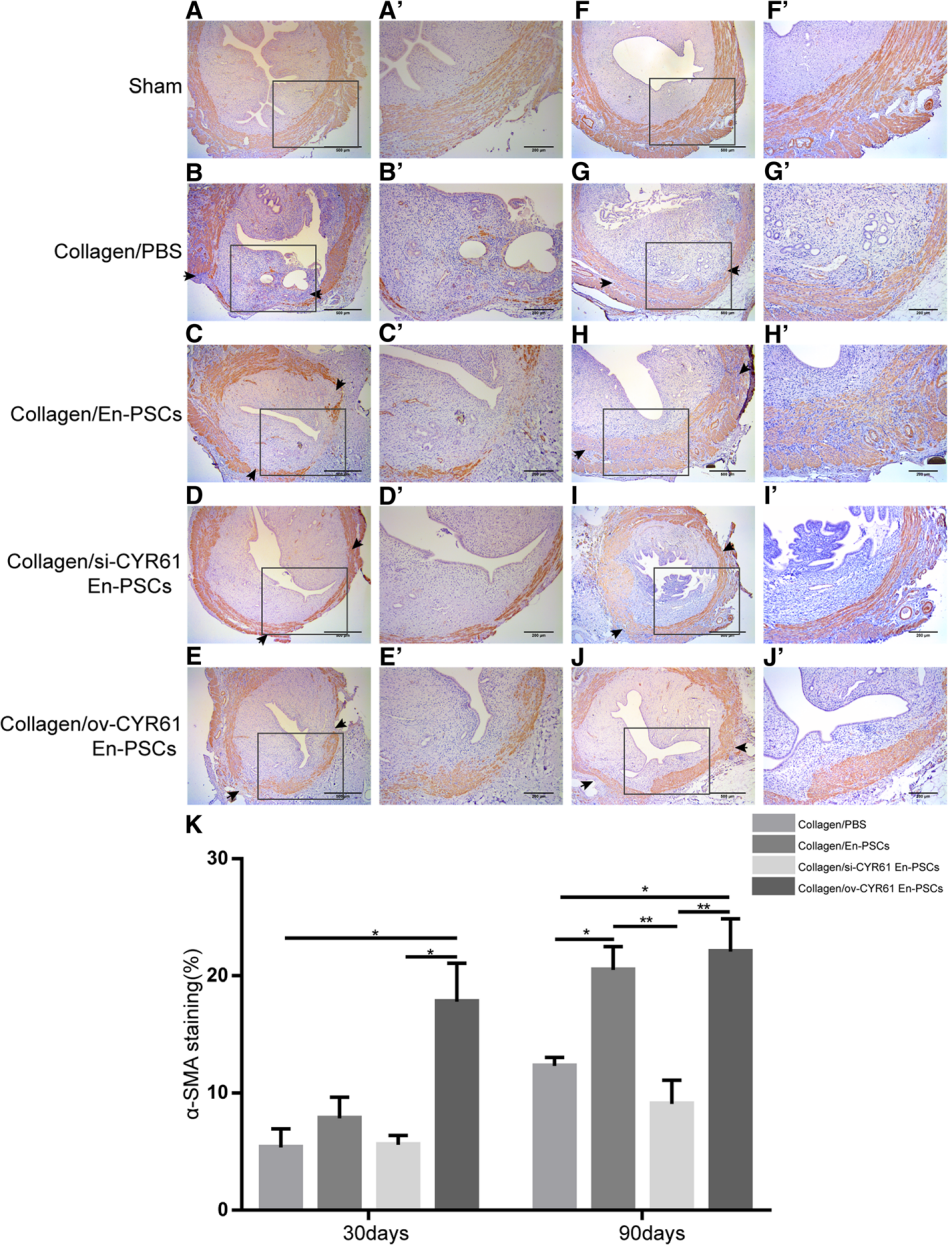
Immunohistochemical staining of α-SMA for smooth muscle regeneration after transplantation of collagen/transfected En-PSCs. Immunohistochemical staining of α-SMA for smooth muscle regeneration at 30 days (**A**–**E**) and 90 days (**F**–**J**) in sham group (**A**, **F**), collagen/PBS group (**B**, **G**), collagen/En-PSCs group (**C**, **H**), collagen/si-CYR61 En-PSCs group (**D**, **I**), and collagen/ov-CYR61 En-PSCs (**E**, **J**). Arrowheads indicate repair sites. Scale bar, 500 μm. Local magnification of figures (**A**–**J**) were showed in the pictures (**A′**–**J′**). Scale bar, 200 μm. **K** The statistical analysis of the ratio of positive area in the regenerative uterine horn to the total area of the uteri. Each experiment was repeated four times. Data were presented as mean ± SEM. *P < 0.05 and **P < 0.01

Uterine smooth muscle bundles at the injured site in rats from each group were detected by a-SMA antibody staining. At 30 days after treatment, relative to the collagen/PBS group, the smooth muscle in the collagen/ov-CYR61 En-PSCs group was more regular (Fig. 4E, E′). The percentage of a-SMA-positive area in the collagen/ov-CYR61 En-PSCs group (17.810% ± 3.246%) was higher than that in the collagen/PBS group (5.332% ± 1.614%) (*P* < 0.05) or the collagen/si-CYR61 En-PSCs group (5.561% ± 0.807%) (*P* < 0.05) (Fig. 4K). At 90 days after treatment, the percentage of a-SMA-positive area in the collagen/ov-CYR61 En-PSCs group (22.062% ± 2.806%) was higher than that in the collagen/si-CYR61 En-PSCs group (9.066% ± 2.020%) (*P* < 0.01) (Fig. 4K).

**Fig. 5:**
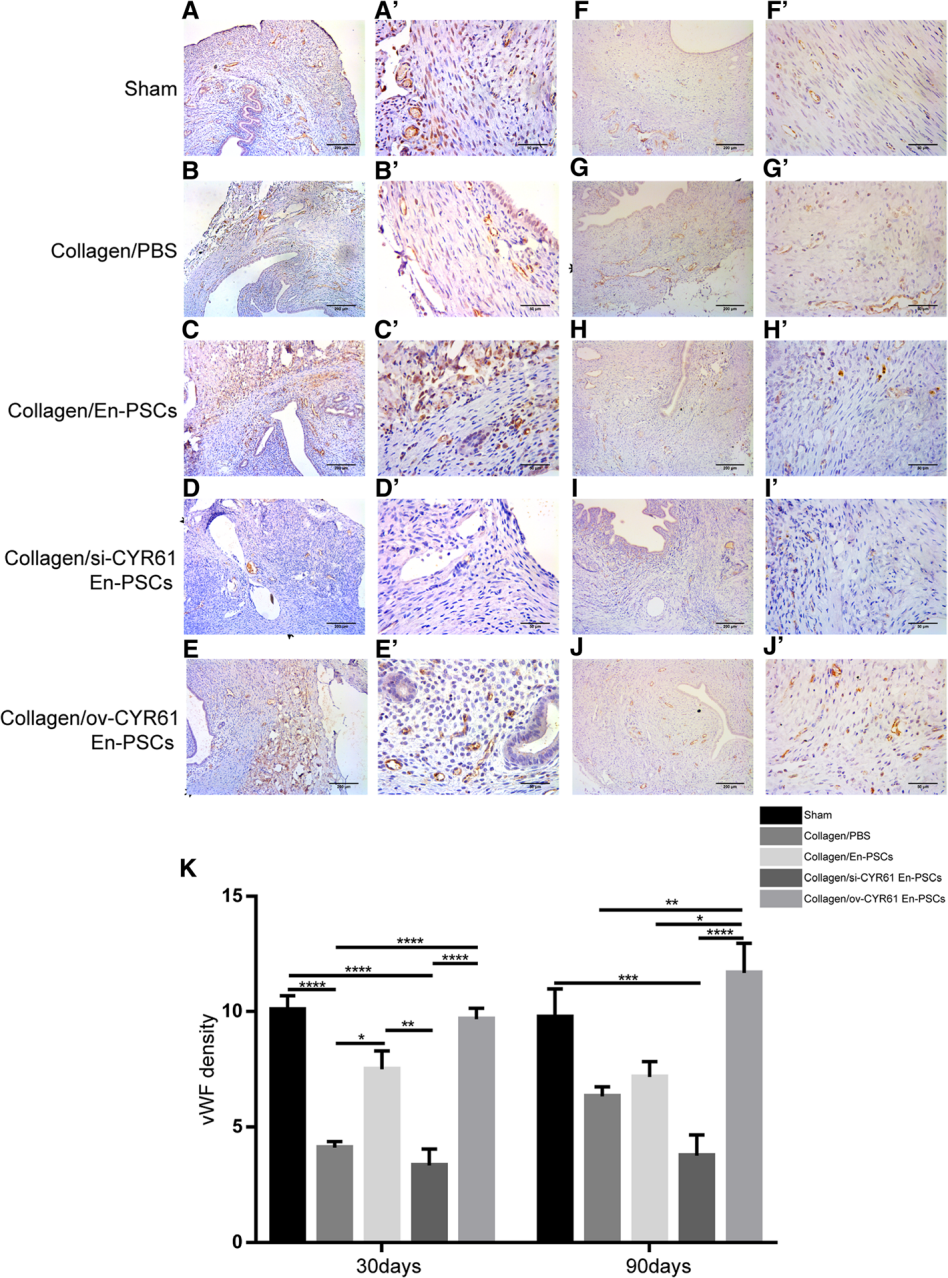
Immunohistochemical staining of vWF in the newly regenerated uteri after transplantation of collagen/transfected En-PSCs. Detection of vWF expression for blood vessels in the regenerated uteri at days 30 days (**A**–**E**) and 90 days (**F**–**I**) in sham group (**A**, **F**), collagen/PBS group (**B**, **G**), collagen/si-CYR61 En-PSCs group (**C**, **H**), collagen/vector En-PSCs group (**D**, **I**), and collagen/ov-CYR61 En-PSCs group (**E**, **J**). Arrowheads indicate repair site. Scale bar, 500 μm. Local magnification of figures (**A**–**J**) were showed in the pictures (**A′**–**J′**). Scale bar, 50 μm. **K** The statistical analysis of the capillary vessels number from at least three randomly selected fields under a magnification of 400. Each experiment was repeated four times. Data were presented as mean ± SEM. *P < 0.05, **P < 0.01, ***P < 0.001, and ****P < 0.0001

At 30 days after treatment, compared with the collagen/PBS group and the collagen/si-CYR61 En-PSCs group, the collagen/ov-CYR61 En-PSCs group induced more blood vessels and these vessels were more evenly distributed (Fig. 5E, E′). The density of blood vessels in the collagen/ov-CYR61 En-PSCs group (9.667 ± 0.482) was greater than that in the collagen/PBS group (4.111 ± 0.261) (*P* < 0.0001) or the collagen/si-CYR61 En-PSCs group (3.333 ± 0.707) (*P* < 0.0001) (Fig. 5K). At 90 days after treatment, the distribution of blood vessels in the collagen/ov-CYR61 En-PSCs group (Fig. 5J, J′) was similar to that in the sham group (Fig. 5F, F′), the blood vessel distribution in the collagen/PBS group (Fig. 5G, G′) was disordered, and in the collagen/si-CYR61 En-PSCs group (Fig. 5I, I′), there were few blood vessels. Blood vessel density in the collagen/ov-CYR61 En-PSCs group (11.667 ± 1.287) was greater than that in the collagen/En-PSCs group (7.167 ± 0.672) (*P* < 0.05) or the collagen/si-CYR61 En-PSCs group (3.750 ± 0.906) (*P* < 0.0001) (Fig. 5K).

### Regenerated uterine fertility assessment

**Fig. 6:**
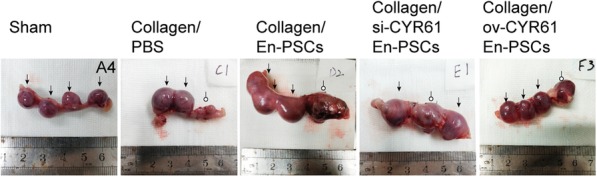
Embryos implantation of different groups after transplantation of collagen/transfected En-PSCs. Pregnancies of different groups at 90 days postoperative in sham group, collagen/PBS and collagen/En-PSCs group, collagen/si-CYR61 En-PSCs, and collagen/ov-En-PSCs. Hollow circle arrows indicated embryos implanted in the injured site. Black arrows showed embryos in the normal tissue

At 90 days after surgical treatment, a fertility assessment revealed the presence of viable embryos in some of the regenerated uteri (Fig. 6). Indeed, pregnancy rates (Table 2) were 100% in the sham group, 40% in the collagen/PBS group, 80% in the collagen/En-PSCs group, 12.5% in the collagen/si-CYR61 En-PSCs group, and 80% in the collagen/ov-CYR61 En-PSCs group. At the injured site, there were no embryos evident in rats of the collagen/PBS group, two embryos in the collagen/En-PSCs group, one embryo in the collagen/si-CYR61 En-PSCs group, and four embryos in the collagen/ov-CYR61 En-PSCs group.

**Table 2:** Reproductive outcomes comparison among different treatments for 90 days after uterine injury

Variable	Total number of uterine horns	Non-pregnant uterine horns (%)	Pregnant uterine horns (%)	Uterine horns with embryo implantation on the scar site (%)
Sham	10	0	10 (100)	
Collagen/PBS	10	6 (60)	4 (40)	0 (0)
Collagen/En-PSCs	10	2 (20)	8 (80)	2 (20)
Collagen/Si-CYR61 En-PSCs	8	7 (87.5)	1 (12.5)	1 (12.5)
Collagen/ov-CYR61 En-PSCs	10	2 (20)	8 (80)	4 (40)
*P* value			0.009	

^a^ Each rat has two uterine horns

^b^ Bonferroni correction: critical level of significance, *p* < 0.008. There was no statistical difference between two groups

## Discussion

Tissue repair often requires angiogenesis to support tissue vascularization and regeneration [30]. The present study suggested that collagen/ov-CYR61 En-PSCs induced angiogenesis and improved the repair of full-thickness uterine injury in rats, restoring both structure and function of injured uteri. These genetically modified En-PSCs might provide the new knowledge for endometrial regeneration.

MSCs express specific markers like CD73, CD90, and CD105 and were negative for hematopoietic markers such as CD34, CD45, and HLA-DR [29]. Simultaneously, they possess multipotency differentiating in vitro into adipocytes [19], osteoblasts, chondrocytes [31], phagocytes, and granulocytes [19]. CD146+PDGFRβ+ human endometrial cells are a small population of MSC-like cells that may be responsible for endometrial cyclical growth, named as eMSCs [10]. In the present study, sorted En-PSCs (CD146+CD34-CD45-CD56-CD144-) not only expressed MSCs markers but also possessed multilineage differentiation potency. En-PSCs expressing PDGFRβ may be one subset of eMSCs. Firstly, the proliferation rate of eMSCs is greater than that of bone marrow-derived and dental pulp stem cells [32] and stem cells derived from umbilical cord [33]. Secondly, eMSCs show superior immunomodulatory effect compared with bone marrow-derived stem cells [34]. Thirdly, En-PSCs expressing PDGFRβ, NG2, and α-SMA, as perivascular cells, are able to maintain endothelial tube networks and improve angiogenic sprouting in vitro [19].

In response to injury, MSCs secrete large quantities of cytokines that are promoting angiogenesis, supporting of growth and differentiation of local cells and anti-scarring to regenerate the tissues [35]. LC-MS/MS identified that En-PSCs secreted angiogenesis-related factors such as connective tissue growth factor (CTGF), Angiopoietin-1 (Ang1), and CYR61. CTGF-null mice show defects in angiogenesis, with impaired interaction between endothelial cells and perivascular cells and collagen IV deficiency in the endothelial basement membrane [36]. Previous study showed prominent defects in endocardial and myocardial development as well as a less complex vascular network in mice embryos were observed in a knockout model of ANGPT1 [37, 38]. Due to severe defects in cardiac perivalvular morphogenesis, impaired placentation, and loss of vascular integrity, CYR61-null mice are embryonic lethal [21, 39]. CYR61 binds to integrin αvβ3 to support endothelial cell adhesion [40]. Moreover, CYR61 regulates Dll4 expression, Dll-Notch signaling, and vascular endothelial growth factor receptor 2 (VEGFR2) signaling, thus regulating the interaction between tip and stalk cells to form the endothelial lumen of the vessel [41–43]. Furthermore, CYR61 induces IL-6 expression in macrophages through integrin αMβ2 and promotes intestinal epithelial cell proliferation in fibroblasts through α6β1 [44]. The present study found that CYR61 promote HUVECs to form tubes while inhibition of CYR61 can inhibit tube formation. As expected, En-PSCs were higher in promoting migration than si-NC En-PSCs and lower than ov-CYR61 En-PSCs. CYR61 content in En-PSCs supernatant was higher than si-CYR61 En-PSCs and lower than ov-CYR61 En-PSCs. In vivo study showed that ov-CYR61 En-PSCs induced increased expression of vWF and promote endometrial repair compared with En-PSCs. Therefore, different angiogenic properties of En-PSCs, si-CYR61 En-PSCs, and ov-CYR61 En-PSCs can be explained by different levels of expression of CYR61 in these cells. We also detected the presence of αvβ3 as a potential factor relevant to angiogenesis (data not shown) and hypothesized that CYR61/αvβ3 was an important pathway for the angiogenic process. However, the present data is not enough to explain the molecular mechanism in occurrence of uterine neovascularization. Further studies will be needed to fully clarify the mechanism of angiogenesis induced by CYR61 secreted by En-PSCs in uteri.

In tissue engineering, selection of a suitable source of stem cells is highly important for regeneration. Functional regeneration of injured rat uteri has been realized by transplantation of human basic fibroblast growth factor, bone marrow mesenchymal stem cells, or umbilical cord-derived mesenchymal stem cells with collagen scaffolds [6, 27, 28]. A suitable cell source must have the following characteristics and advantages: not carrying the risk of disease transmission, inducing angiogenesis, not triggering host immune, and producing an extracellular matrix [45, 46]. Hence, genetically modified En-PSCs are expected to be used successfully in endometrial tissue regeneration.

## Conclusions

In the present study, it has been identified that En-PSCs induce angiogenesis via CYR61. The current data highlight the importance of collagen/ov-CYR61 En-PSCs system, promoting regeneration of endometrium and myometrium, inducing angiogenesis in rat uterine injury, and improving pregnancy outcomes. In addition, the remarkable observation is that transplantation of ov-CYR61 En-PSCs increased the number of embryos implanted at injured sites in rat.

## Additional files

Additional file 1: **Figure S1.** En-PSCs with different CYR61 expression levels. A CYR61 was detected by Western Blot in transfected En-PSCs. B CCK-8 assay was used to detect the proliferation of transfected En-PSCs. C Wound-healing assay was used to detect the migration effect of En-PSCs supernatant on ESCs. Scale bar, 500 μm. D The percentage of cell migration was detected in wound-healing experiments after 24 h. E CCK-8 assay was used to detect the proliferation of En-PSCs supernatant on the ESCs. Bars represent the means ± S.E.M. of three independent experiments performed in triplicate. Data were presented as mean + SEM. *P < 0.05, **P < 0.01, ***P < 0.001, and ****P < 0.0001. (TIF 8624 kb)

Additional file 2: **Figure S2.** Morphology of uterine injury following different treatments. Gross view of uterine injures at days 30 and 90 post-transplantation in sham group, collagen/PBS group, the collagen/En-PSCs group, the collagen/si-CYR61 En-PSCs group and the collagen/ov-CYR61 En-PSCs group. (TIF 5149 kb)

Additional file 3: **Table S3.** Secreted protein in En-PSCs supernatant. (DOCX 52 kb)

## Abbreviations

Ang1: Angiopoietin-1
ATRA: All-trans-retinoic acid
bFGF: Fibroblast growth factor-basic
BSA: Bovine serum albumin
CCN1: CCN family member 1
CTGF: Connective tissue growth factor
CYR61: Cysteine-rich angiogenic inducer 61
DAPI: 4′,6-Diamidino-2-phenylindole
ECM: Extracellular matrix
eMSC: Endometrial MSC
En-PSCs: Endometrial perivascular cells
ECM: Extracellular matrix
ESCs: Endometrial stromal cells
FBS: Fetal bovine serum
FITC: Fluorescein isothiocyanate
HUVECs: Human umbilical vein endothelial cells
NF-M: Neurofilament medium poclypeptide
NSE: Neuron-specific enolase
PBS: Phosphate-buffered saline
PE: Phycoerythrin
SD: Sprague-Dawley
SEM: Standard error of the mean
MSCs: Mesenchymal stem cells
VEGF: Vascular endothelial growth factor
VEGFR2: Vascular endothelial growth factor receptor 2
vWF: von Willebrand factor
α-SMA: α-Smooth muscle actin
