# Supplementary figures and images for: Transplantation of human endometrial perivascular cells with elevated CYR61 expression induces angiogenesis and promotes repair of a full-thickness uterine injury in rat
Source: Stem Cell Res Ther. 2019 Jun 18;10:179. doi: 10.1186/s13287-019-1272-3 (PMC6582612; doi:10.1186/s13287-019-1272-3)

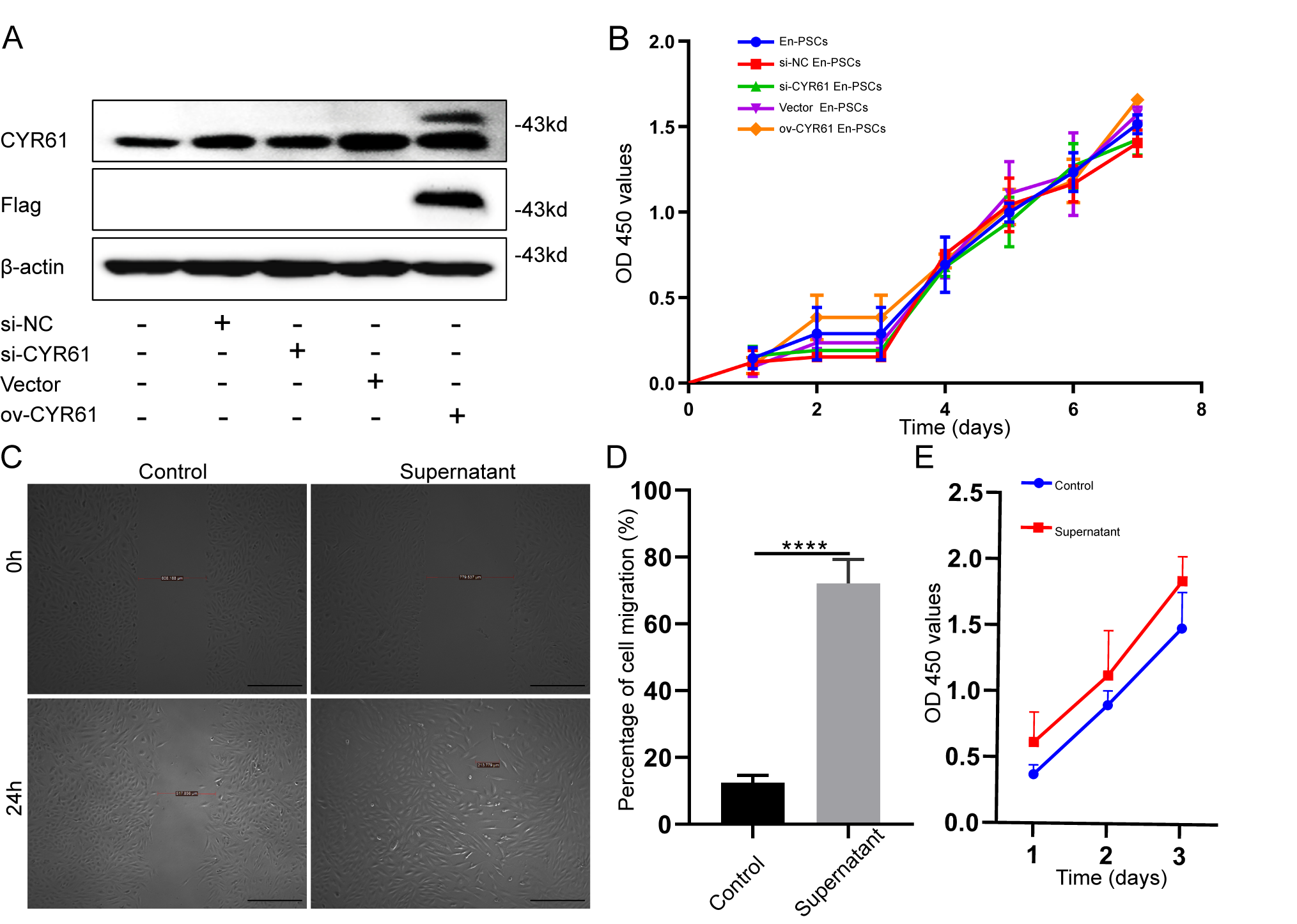

Supplement: Supplementary file 1 — Figure S1. En-PSCs with different CYR61 expression levels. A CYR61 was detected by Western Blot in transfected En-PSCs. B CCK-8 assay was used to detect the proliferation of transfected En-PSCs. C Wound-healing assay was used to detect the migration effect of En-PSCs supernatant on ESCs. Scale bar, 500 μm. D The percentage of cell migration was detected in wound-healing experiments after 24 h. E CCK-8 assay was used to detect the proliferation of En-PSCs supernatant on the ESCs. Bars represent the means ± S.E.M. of three independent experiments performed in triplicate. Data were presented as mean + SEM. *P < 0.05, **P < 0.01, ***P < 0.001, and ****P < 0.0001. (TIF 8624 kb) [file 13287_2019_1272_MOESM1_ESM.tif]

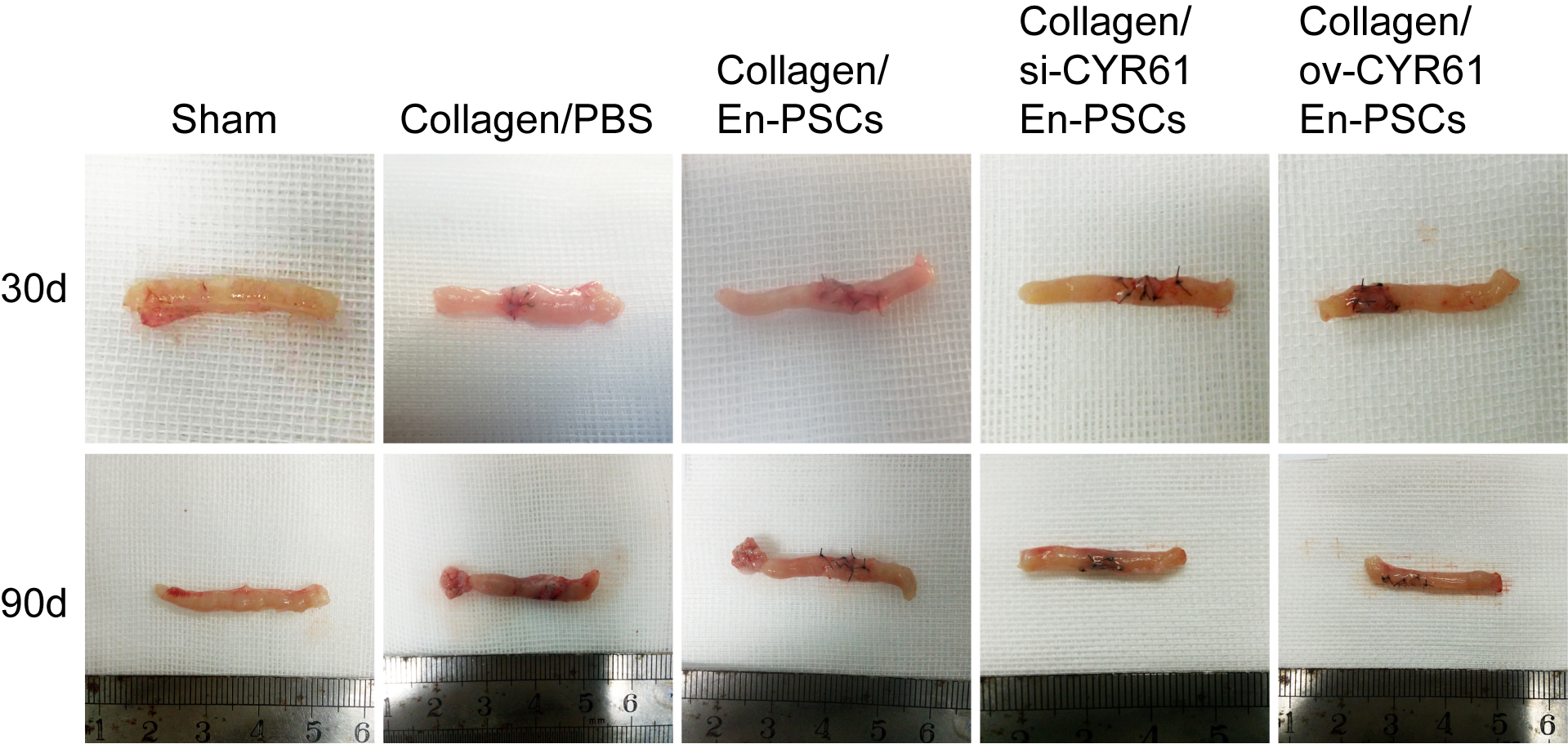

Supplement: Supplementary file 2 — Figure S2. Morphology of uterine injury following different treatments. Gross view of uterine injures at days 30 and 90 post-transplantation in sham group, collagen/PBS group, the collagen/En-PSCs group, the collagen/si-CYR61 En-PSCs group and the collagen/ov-CYR61 En-PSCs group. (TIF 5149 kb) [file 13287_2019_1272_MOESM2_ESM.tif]
