# Supplementary material for: Transplantation of human endometrial perivascular cells with elevated CYR61 expression induces angiogenesis and promotes repair of a full-thickness uterine injury in rat
Source: Stem Cell Res Ther. 2019 Jun 18;10:179. doi: 10.1186/s13287-019-1272-3 (PMC6582612; doi:10.1186/s13287-019-1272-3)
Supplement: Supplementary file 3 — Table S3. Secreted protein in En-PSCs supernatant. (DOCX 52 kb) [file 13287_2019_1272_MOESM3_ESM.docx]

| **Table S3.** Secreted protein in En-PSCs supernatant. | | | | | | |
| --- | --- | --- | --- | --- | --- | --- |
| Protein name | Accession | Score | Mass | Matches | Sequences | emPAI |
| CYR61 | O00622 | 95 | 44165 | 5 (2) | 5 (2) | 0.16 |
| Collagen alpha-2(I) chain | A0A087WTA8 | 10868 | 129586 | 574 (421) | 92 (67) | 36.09 |
| Neuroblastoma suppressor of tumorigenicity 1 | A0A087WTY6 | 31 | 23907 | 3 (3) | 1 (1) | 0.14 |
| Inter-alpha-trypsin inhibitor heavy chain H3 | A0A087WW43 | 59 | 75316 | 15 (3) | 11 (2) | 0.09 |
| Laminin subunit alpha-2 | A0A087WYF1 | 94 | 352491 | 38 (4) | 30 (3) | 0.03 |
| Collagen alpha-6(IV) chain | A0A087WZY5 | 34 | 162427 | 31 (2) | 16 (1) | 0.04 |
| Collagen alpha-1(XV) chain | A0A087X0K0 | 151 | 140428 | 11 (7) | 8 (6) | 0.15 |
| Collagen alpha-1(VI) chain | A0A087X0S5 | 3374 | 109355 | 191 (130) | 54 (36) | 7.11 |
| Laminin subunit alpha-4 | A0A0A0MQS9 | 1551 | 205064 | 125 (74) | 65 (46) | 1.5 |
| Immunoglobulin heavy constant gamma 1 | A0A0A0MS07 | 42 | 32476 | 3 (1) | 2 (1) | 0.1 |
| Gelsolin | A0A0A0MS51 | 885 | 82759 | 59 (35) | 31 (17) | 1.74 |
| Poliovirus receptor | A0A0A0MSA9 | 39 | 43425 | 4 (1) | 2 (1) | 0.08 |
| von Willebrand factor type A | A0A0A0MSD0 | 201 | 404982 | 46 (12) | 35 (11) | 0.1 |
| Glucose-6-phosphate isomerase | A0A0A0MTS2 | 468 | 65012 | 41 (24) | 22 (15) | 1.82 |
| Serum albumin | A0A0C4DGB6 | 392 | 71177 | 61 (19) | 21 (13) | 1.15 |
| Complement C4-B | A0A0G2JL54 | 42 | 189080 | 15 (1) | 14 (1) | 0.02 |
| Alpha-2-antiplasmin | A0A0G2JPA8 | 41 | 48135 | 12 (1) | 8 (1) | 0.07 |
| Renin | A0A1B0GUZ2 | 73 | 40820 | 6 (1) | 5 (1) | 0.08 |
| Cathepsin D | A0A1B0GV23 | 427 | 44316 | 24 (16) | 14 (8) | 0.78 |
| SSc5D | A1L4H1 | 152 | 168091 | 17 (11) | 12 (6) | 0.12 |
| Periostin | B1ALD9 | 1285 | 90714 | 76 (54) | 33 (27) | 3.14 |
| Insulin-like growth factor binding protein 3 | B3KWK7 | 68 | 22229 | 4 (2) | 4 (2) | 0.32 |
| Fibulin-7 | B8ZZC1 | 34 | 41008 | 3 (2) | 2 (1) | 0.08 |
| Prosaposin | C9JIZ6 | 164 | 60227 | 24 (10) | 15 (7) | 0.45 |
| Cartilage-associated protein | C9JP16 | 63 | 41920 | 3 (2) | 3 (2) | 0.16 |
| Target of Nesh-SH3 | D3YTG3 | 179 | 195888 | 19 (5) | 15 (4) | 0.09 |
| Vitamin D-binding protein | D6RF35 | 184 | 54583 | 14 (6) | 10 (4) | 0.34 |
| Collagen alpha-1(XII) chain | D6RGG3 | 5304 | 334193 | 399 (261) | 147 (109) | 4.41 |
| Lactotransferrin | E7EQB2 | 48 | 78459 | 11 (2) | 8 (2) | 0.09 |
| Neuropilin | E9PEP6 | 196 | 102487 | 23 (10) | 17 (7) | 0.29 |
| Plasma protease C1 inhibitor | E9PGN7 | 348 | 59683 | 30 (18) | 15 (7) | 0.54 |
| CD59 glycoprotein | E9PNW4 | 68 | 12604 | 10 (5) | 2 (2) | 1.05 |
| EGF-containing fibulin-like extracellular matrix protein 2 | E9PRU1 | 165 | 50555 | 12 (8) | 6 (3) | 0.29 |
| Collagen alpha-1(XXI) chain | F5GZK2 | 22 | 100032 | 18 (1) | 12 (1) | 0.03 |
| Tenascin | F5H7V9 | 200 | 206659 | 17 (7) | 15 (5) | 0.08 |
| Dickkopf-related protein 3 | F6SYF8 | 299 | 41121 | 23 (16) | 10 (8) | 1.17 |
| Tetraspanin | F8VWK8 | 115 | 24092 | 12 (5) | 7 (2) | 0.3 |
| Apolipoprotein A-I | F8W696 | 57 | 28005 | 3 (2) | 2 (1) | 0.25 |
| NPC intracellular cholesterol transporter 2 | G3V3E8 | 25 | 19560 | 9 (1) | 8 (1) | 0.17 |
| Latent-transforming growth factor beta-binding protein 2 | G3V3X5 | 649 | 198942 | 55 (29) | 27 (15) | 0.38 |
| Fibulin-5 | G3V4U0 | 102 | 53242 | 14 (5) | 9 (4) | 0.27 |
| Laminin subunit beta-1 | G3XAI2 | 1486 | 207703 | 95 (53) | 57 (31) | 0.89 |
| Protein S | G5E9F8 | 25 | 61914 | 2 (1) | 2 (1) | 0.05 |
| Syndecan-1 | H7C1K4 | 60 | 22100 | 4 (4) | 1 (1) | 0.15 |
| Alpha-fetoprotein | J3KMX3 | 136 | 72286 | 10 (4) | 7 (3) | 0.14 |
| Twisted gastrulation protein homolog 1 | J3QS03 | 24 | 21321 | 2 (2) | 1 (1) | 0.16 |
| Microfibril-associated glycoprotein 4 | K7ES70 | 252 | 31648 | 16 (11) | 8 (4) | 1.46 |
| Alpha-1B-glycoprotein | M0R009 | 43 | 33720 | 3 (2) | 2 (1) | 0.1 |
| Sulfhydryl oxidase 1 | O00391 | 1222 | 83324 | 79 (57) | 40 (29) | 3.86 |
| Agrin | O00468 | 445 | 225246 | 34 (22) | 25 (15) | 0.3 |
| Syntenin-1 | O00560 | 223 | 32595 | 14 (8) | 7 (4) | 0.79 |
| Immunoglobulin superfamily containing leucine-rich repeat protein | O14498 | 239 | 46596 | 17 (12) | 8 (7) | 0.98 |
| ANG-2 | O15123 | 34 | 57396 | 29 (1) | 13 (1) | 0.06 |
| Laminin subunit alpha-5 | O15230 | 1412 | 412023 | 96 (52) | 65 (35) | 0.36 |
| Calumenin | O43852 | 336 | 37198 | 30 (17) | 20 (12) | 2.6 |
| EGF-like repeat and discoidin I-like domain-containing protein 3 | O43854 | 70 | 55098 | 20 (6) | 12 (4) | 0.34 |
| Neuropilin-2 | O60462 | 32 | 106132 | 8 (2) | 6 (1) | 0.06 |
| Gremlin-1 | O60565 | 229 | 21197 | 18 (8) | 12 (4) | 1.8 |
| Sushi repeat-containing protein SRPX2 | O60687 | 119 | 54192 | 14 (4) | 10 (3) | 0.19 |
| Stanniocalcin-2 | O76061 | 216 | 34083 | 20 (10) | 11 (8) | 1.53 |
| Serine protease 23 | O95084 | 34 | 43487 | 7 (3) | 5 (2) | 0.25 |
| Coagulation factor X | P00742 | 172 | 56065 | 19 (7) | 8 (4) | 0.41 |
| Tissue-type plasminogen activator | P00750 | 265 | 65043 | 15 (8) | 10 (5) | 0.28 |
| Antithrombin-III | P01008 | 62 | 53025 | 15 (3) | 13 (2) | 0.2 |
| Alpha-2-macroglobulin | P01023 | 842 | 164613 | 68 (40) | 45 (29) | 0.87 |
| Complement C3 | P01024 | 73 | 188569 | 83 (5) | 18 (4) | 0.07 |
| Cystatin-C | P01034 | 305 | 16017 | 25 (12) | 5 (4) | 2.17 |
| Cystatin-SN | P01037 | 150 | 16605 | 7 (4) | 3 (2) | 0.45 |
| Transforming growth factor beta-1 proprotein | P01137 | 98 | 44997 | 7 (2) | 4 (1) | 0.07 |
| Insulin-like growth factor II | P01344 | 78 | 20583 | 3 (2) | 2 (1) | 0.16 |
| Collagen alpha-1(III) chain | P02461 | 5363 | 139733 | 420 (272) | 94 (63) | 15.39 |
| Collagen alpha-1(IV) chain | P02462 | 682 | 161654 | 74 (33) | 27 (13) | 0.55 |
| Apolipoprotein E | P02649 | 67 | 36246 | 7 (2) | 6 (2) | 0.19 |
| Fibronectin | P02751 | 5713 | 266052 | 389 (259) | 106 (76) | 5.14 |
| Alpha-2-HS-glycoprotein | P02765 | 72 | 40098 | 6 (2) | 5 (2) | 0.17 |
| Serotransferrin | P02787 | 30 | 79294 | 6 (1) | 6 (1) | 0.04 |
| Interstitial collagenase | P03956 | 743 | 54144 | 51 (35) | 23 (18) | 2.89 |
| Vitronectin | P04004 | 53 | 55069 | 10 (3) | 8 (2) | 0.12 |
| Annexin A1 | P04083 | 372 | 38918 | 20 (15) | 15 (11) | 1.66 |
| Plasminogen activator inhibitor 2 | P05120 | 458 | 46851 | 23 (13) | 13 (7) | 0.72 |
| Plasminogen activator inhibitor 1 | P05121 | 1157 | 45088 | 70 (47) | 21 (16) | 5.75 |
| Plasma serine protease inhibitor | P05154 | 28 | 45760 | 5 (2) | 3 (1) | 0.07 |
| Collagen alpha-2(V) chain | P05997 | 2038 | 145790 | 172 (110) | 61 (39) | 3.04 |
| Protein S100-A9 | P06702 | 52 | 13291 | 8 (4) | 6 (2) | 0.58 |
| Glia-derived nexin | P07093 | 471 | 44202 | 23 (17) | 18 (13) | 1.55 |
| Annexin A2 | P07355 | 712 | 38808 | 51 (29) | 25 (16) | 5.56 |
| PG-S2 | P07585 | 385 | 40064 | 30 (21) | 15 (10) | 2.04 |
| Cathepsin B | P07858 | 598 | 38766 | 33 (21) | 10 (8) | 2.42 |
| MMP-2 | P08253 | 3054 | 74918 | 181 (121) | 43 (32) | 12.09 |
| Stromelysin-1 | P08254 | 358 | 54228 | 34 (21) | 21 (15) | 2.06 |
| Inhibin beta A chain | P08476 | 156 | 48210 | 19 (7) | 10 (4) | 0.39 |
| Collagen alpha-2(IV) chain | P08572 | 2285 | 168646 | 180 (110) | 65 (45) | 2.9 |
| Complement factor H | P08603 | 42 | 143680 | 18 (1) | 16 (1) | 0.02 |
| Galectin-1 | P09382 | 424 | 15048 | 24 (19) | 11 (9) | 20.03 |
| High mobility group protein B1 | P09429 | 237 | 25049 | 20 (14) | 10 (7) | 2.08 |
| SPARC | P09486 | 618 | 35465 | 76 (37) | 18 (13) | 12.3 |
| Serglycin | P10124 | 37 | 17812 | 13 (1) | 6 (1) | 0.19 |
| Thioredoxin | P10599 | 98 | 12015 | 8 (3) | 5 (3) | 1.13 |
| Tissue factor pathway inhibitor | P10646 | 120 | 36075 | 11 (5) | 6 (3) | 0.42 |
| Clusterin | P10909 | 85 | 53031 | 20 (7) | 12 (5) | 0.35 |
| Hyaluronan and proteoglycan link protein 1 | P10915 | 29 | 40767 | 6 (1) | 6 (1) | 0.08 |
| Laminin B2 chain | P11047 | 1387 | 183191 | 86 (53) | 57 (32) | 0.99 |
| Collagen alpha-2(VI) chain | P12110 | 923 | 109709 | 82 (45) | 40 (25) | 1.8 |
| Collagen alpha-3(VI) chain | P12111 | 6407 | 345167 | 380 (272) | 168 (132) | 5.13 |
| BMP-1 | P13497 | 72 | 113516 | 16 (7) | 11 (5) | 0.15 |
| Versican core protein | P13611 | 479 | 374585 | 40 (23) | 21 (9) | 0.13 |
| Nidogen-1 | P14543 | 1393 | 139142 | 113 (76) | 45 (36) | 2.42 |
| TIMP-2 | P16035 | 574 | 25067 | 34 (25) | 18 (14) | 11.19 |
| Carboxypeptidase E | P16870 | 38 | 53516 | 9 (1) | 6 (1) | 0.06 |
| Galectin-3 | P17931 | 258 | 26193 | 21 (12) | 5 (5) | 1.05 |
| IBP-2 | P18065 | 316 | 35875 | 20 (11) | 13 (7) | 1.03 |
| Peptidyl-glycine alpha-amidating monooxygenase | P19021 | 274 | 109119 | 27 (20) | 17 (10) | 0.47 |
| Follistatin | P19883 | 32 | 40148 | 1 (1) | 1 (1) | 0.08 |
| Biglycan | P21810 | 522 | 42027 | 31 (23) | 15 (12) | 1.48 |
| IBP-4 | P22692 | 106 | 29113 | 9 (6) | 5 (3) | 0.92 |
| FIBL-1 | P23142 | 383 | 81268 | 26 (17) | 15 (12) | 0.74 |
| IBP-6 | P24592 | 155 | 26219 | 6 (5) | 3 (3) | 0.82 |
| IBP-5 | P24593 | 80 | 31576 | 10 (5) | 5 (2) | 0.22 |
| TSG-14 | P26022 | 220 | 42519 | 12 (7) | 7 (4) | 0.57 |
| HMG-2 | P26583 | 39 | 24190 | 12 (2) | 7 (2) | 0.3 |
| Collagen alpha-1(VIII) chain | P27658 | 197 | 73431 | 47 (20) | 25 (14) | 0.93 |
| CRP55 | P27797 | 662 | 48283 | 73 (47) | 23 (14) | 3.88 |
| Protein-lysine 6-oxidase | P28300 | 47 | 47599 | 7 (2) | 5 (1) | 0.07 |
| CCN2 | P29279 | 76 | 40289 | 9 (4) | 6 (2) | 0.17 |
| Protein S100-A7 | P31151 | 92 | 11578 | 5 (2) | 5 (2) | 0.69 |
| Guanylate-binding protein 1 | P32455 | 41 | 68401 | 15 (3) | 11 (2) | 0.1 |
| Ribonuclease 4 | P34096 | 94 | 17286 | 7 (5) | 4 (3) | 1.04 |
| Glypican-1 | P35052 | 614 | 62724 | 37 (21) | 20 (12) | 1.26 |
| Fibrillin-1 | P35555 | 525 | 332664 | 76 (32) | 45 (23) | 0.25 |
| Chitinase-3-like protein 1 | P36222 | 33 | 42998 | 9 (1) | 8 (1) | 0.08 |
| PEDF | P36955 | 985 | 46454 | 38 (25) | 20 (14) | 3.22 |
| Collagen alpha-1(XVIII) chain | P39060 | 209 | 179389 | 18 (9) | 11 (4) | 0.09 |
| Prostaglandin-H2 D-isomerase | P41222 | 126 | 21243 | 11 (6) | 8 (4) | 1.09 |
| Biotinidase | P43251 | 114 | 62006 | 11 (4) | 8 (3) | 0.17 |
| Nicotinamide phosphoribosyltransferase | P43490 | 298 | 55772 | 27 (14) | 19 (10) | 0.88 |
| Lumican | P51884 | 307 | 38747 | 27 (14) | 14 (7) | 1.27 |
| Stanniocalcin-1 | P52823 | 120 | 28231 | 8 (3) | 7 (2) | 0.25 |
| Coatomer subunit alpha | P53621 | 158 | 139797 | 26 (6) | 19 (5) | 0.15 |
| Phospholipid transfer protein | P55058 | 285 | 54933 | 20 (7) | 11 (6) | 0.42 |
| Laminin subunit beta-2 | P55268 | 146 | 202982 | 23 (6) | 19 (4) | 0.07 |
| Neutrophil defensin 1 | P59665 | 26 | 10536 | 1 (1) | 1 (1) | 0.33 |
| Beta-2-microglobulin | P61769 | 130 | 13820 | 16 (10) | 8 (6) | 6.23 |
| Peptidyl-prolyl cis-trans isomerase A | P62937 | 600 | 18229 | 34 (30) | 11 (11) | 34.55 |
| Nuclease-sensitive element-binding protein 1 | P67809 | 420 | 35903 | 18 (13) | 11 (8) | 1.64 |
| Dermcidin | P81605 | 130 | 11391 | 11 (7) | 6 (5) | 2.73 |
| HSPG | P98160 | 1080 | 479253 | 74 (44) | 49 (27) | 0.23 |
| Pregnancy-specific beta-1-glycoprotein 6 | Q00889 | 200 | 49182 | 5 (4) | 3 (2) | 0.14 |
| Collagen alpha-1(VII) chain | Q02388 | 586 | 296010 | 58 (24) | 47 (20) | 0.26 |
| Collagen alpha-1(XIV) chain | Q05707 | 950 | 194478 | 73 (38) | 45 (27) | 0.59 |
| Mitochondrial matrix protein p32 | Q07021 | 32 | 31742 | 1 (1) | 1 (1) | 0.1 |
| Collagen alpha-1(XVI) chain | Q07092 | 68 | 159477 | 20 (2) | 13 (1) | 0.02 |
| Galectin-3-binding protein | Q08380 | 858 | 66202 | 54 (36) | 27 (20) | 2.7 |
| Lactadherin | Q08431 | 310 | 43894 | 27 (16) | 15 (9) | 1.39 |
| Polypeptide N-acetylgalactosaminyltransferase 2 | Q10471 | 148 | 65433 | 21 (4) | 17 (3) | 0.22 |
| Follistatin-related protein 1 | Q12841 | 389 | 36103 | 27 (17) | 15 (10) | 2.42 |
| Prolyl endopeptidase FAP | Q12884 | 38 | 88341 | 7 (1) | 7 (1) | 0.04 |
| Pappalysin-1 | Q13219 | 74 | 185645 | 11 (3) | 9 (2) | 0.04 |
| Myeloma cell metalloproteinase | Q13443 | 58 | 93006 | 11 (4) | 7 (3) | 0.11 |
| CD166 antigen | Q13740 | 119 | 65745 | 7 (4) | 5 (3) | 0.16 |
| Dystroglycan | Q14118 | 109 | 97723 | 18 (4) | 10 (3) | 0.14 |
| TGF-beta1-BP-1 | Q14766 | 341 | 195112 | 41 (16) | 24 (11) | 0.26 |
| LysRS | Q15046 | 68 | 68461 | 17 (2) | 10 (2) | 0.1 |
| Procollagen C-endopeptidase enhancer 1 | Q15113 | 1297 | 48797 | 77 (48) | 23 (19) | 4.47 |
| Angiopoietin-1 | Q15389 | 204 | 58046 | 21 (11) | 13 (7) | 0.47 |
| Transforming growth factor-beta-induced protein ig-h3 | Q15582 | 1967 | 75261 | 122 (83) | 36 (28) | 11.4 |
| Insulin-like growth factor-binding protein 7 | Q16270 | 770 | 30138 | 51 (38) | 18 (15) | 11.28 |
| Extracellular matrix protein 1 | Q16610 | 723 | 62232 | 74 (41) | 25 (16) | 2.62 |
| Glutaminyl-peptide cyclotransferase | Q16769 | 89 | 40965 | 7 (3) | 6 (2) | 0.17 |
| Prolyl 3-hydroxylase 1 | Q32P28 | 49 | 84196 | 6 (3) | 4 (2) | 0.08 |
| Metalloproteinase inhibitor 1 | Q5H9A7 | 569 | 16560 | 29 (22) | 10 (7) | 10.26 |
| NAD(P)H-hydrate epimerase | Q5T3I4 | 51 | 27011 | 16 (2) | 5 (1) | 0.12 |
| Inter-alpha-trypsin inhibitor heavy chain H2 | Q5T985 | 144 | 105606 | 14 (6) | 11 (4) | 0.13 |
| Vasorin | Q6EMK4 | 232 | 72751 | 19 (12) | 9 (7) | 0.7 |
| Suprabasin | Q6UWP8 | 71 | 60562 | 17 (2) | 13 (2) | 0.11 |
| Inactive serine protease PAMR1 | Q6UXH9 | 420 | 82598 | 46 (26) | 20 (13) | 0.94 |
| Coiled-coil domain-containing protein 80 | Q76M96 | 284 | 108505 | 27 (13) | 17 (8) | 0.35 |
| Four-jointed box protein 1 | Q86VR8 | 33 | 48705 | 7 (1) | 6 (1) | 0.07 |
| Adipocyte enhancer-binding protein 1 | Q8IUX7 | 754 | 131588 | 64 (30) | 38 (18) | 0.8 |
| Signal peptide, CUB and EGF-like domain-containing protein 3 | Q8IX30 | 36 | 114399 | 7 (1) | 7 (1) | 0.03 |
| Programmed cell death 6-interacting protein | Q8WUM4 | 376 | 96590 | 21 (9) | 14 (6) | 0.26 |
| Peroxidasin homolog | Q92626 | 2010 | 167793 | 139 (83) | 65 (43) | 2.06 |
| Serine protease HTRA1 | Q92743 | 269 | 52167 | 30 (17) | 17 (12) | 1.22 |
| Gamma-glutamyl hydrolase | Q92820 | 282 | 36340 | 22 (14) | 12 (7) | 1.01 |
| Myeloid-derived growth factor | Q969H8 | 117 | 18897 | 8 (6) | 5 (4) | 0.93 |
| Collagen triple helix repeat-containing protein 1 | Q96CG8 | 50 | 26777 | 7 (3) | 6 (3) | 0.42 |
| Retinoic acid receptor responder protein 2 | Q99969 | 150 | 18948 | 12 (10) | 5 (4) | 1.67 |
| Plasma alpha-L-fucosidase | Q9BTY2 | 122 | 54374 | 14 (4) | 10 (4) | 0.27 |
| Spondin-2 | Q9BUD6 | 111 | 36337 | 31 (11) | 13 (6) | 1.01 |
| Chitinase domain-containing protein 1 | Q9BWS9 | 156 | 45083 | 15 (5) | 13 (5) | 0.42 |
| EMILIN-2 | Q9BXX0 | 46 | 116869 | 13 (2) | 10 (1) | 0.03 |
| Platelet-derived growth factor D | Q9GZP0 | 99 | 43677 | 3 (2) | 2 (1) | 0.08 |
| Protein Wnt-5b | Q9H1J7 | 33 | 41665 | 12 (1) | 7 (1) | 0.08 |
| Aminopeptidase B | Q9H4A4 | 175 | 73234 | 11 (7) | 7 (4) | 0.19 |
| Matrix-remodeling-associated protein 5 | Q9NR99 | 605 | 314065 | 64 (21) | 46 (11) | 0.12 |
| Olfactomedin-like protein 3 | Q9NRN5 | 488 | 46380 | 29 (18) | 15 (9) | 1.13 |
| LTBP-3 | Q9NS15 | 34 | 146451 | 7 (2) | 6 (2) | 0.05 |
| Exostosin-like 2 | Q9UBQ6 | 88 | 37726 | 14 (5) | 9 (3) | 0.29 |
| Carboxypeptidase A4 | Q9UI42 | 62 | 47550 | 16 (5) | 8 (2) | 0.22 |
| N-acetylglucosamine-1-phosphotransferase subunit gamma | Q9UJJ9 | 59 | 34351 | 3 (2) | 3 (2) | 0.2 |
| Angiopoietin-related protein 2 | Q9UKU9 | 98 | 57582 | 14 (2) | 10 (2) | 0.12 |
| C-type lectin domain family 11 member A | Q9Y240 | 265 | 36015 | 19 (12) | 14 (9) | 1.88 |
| Lysyl oxidase homolog 2 | Q9Y4K0 | 445 | 88778 | 38 (20) | 22 (11) | 0.79 |
| EMILIN-1 | Q9Y6C2 | 540 | 107941 | 44 (28) | 26 (19) | 0.93 |
